# Don’t Hang Around, It Could Be Incidental: A Case Report of Hangman’s Fracture and Review of the Literature

**DOI:** 10.7759/cureus.63285

**Published:** 2024-06-27

**Authors:** Edoardo G Frezza, Manya Bali, Eldo Frezza

**Affiliations:** 1 Medicine, Trinity School of Medicine, Ribishi, VCT; 2 Medicine, California Northstate University College of Medicine, Elk Grove, USA; 3 Surgery, California Northstate University College of Medicine, Elk Grove, USA

**Keywords:** hangman’s fracture, spine injury, ct, axis, spondylolisthesis

## Abstract

Neck injury fractures are commonly associated with high-impact trauma, such as motor vehicle accidents or falls from heights. However, this case underscores that it is possible to sustain such a fracture even from minor falls. As of now, there are no such reported cases. This case report highlights the importance of a thorough medical history when assessing patients with neck pain following falls.

A 59-year-old male experienced a fainting episode after suffering from vomiting and diarrhea, resulting in him hitting his head. The patient attributed his neck pain to a sudden twisting of his neck. The pain originated from the base of his skull, primarily on the left side, extending to the scalp and the left shoulder. After enduring four days of intense pain that limited his ability to rotate his neck and bend to tie his shoes, he sought medical attention and underwent a neck CT scan, which led to the diagnosis of a "hangman's fracture."

This injury was diagnosed in a clinical setting. Healthcare providers should inquire about the circumstances of the fall, the patient's position, associated symptoms, and any relevant pre-existing conditions. This approach ensures an accurate diagnosis and timely treatment. Comprehensive history-taking is essential for identifying high-risk situations and preventing complications that may arise from overlooked minor falls, ultimately enhancing patient safety, especially in cases of neck and spine injuries.

## Introduction

Traumatic spondylolisthesis of the axis, popularly described as “hangman's fracture,” is considered one of the most frequent forms of high cervical spine injury [1]. It is, by definition, a bilateral fracture of the “pars interarticularis” (or isthmus) of the C2 vertebra [2]. The fracture was first described in the 1860s subsequent to judicial hangings, with the proposed mechanisms of hyperextension and distraction at the level of C2-C3, yet in a post-mortem review of 34 hanging victims, less than 10% had this fracture pattern [3]. Nevertheless, Schneider et al. coined the term in an eight-patient case series [4], and it remains a popular term. The most common etiological factor for this injury is a highly traumatic event, such as a motor vehicle accident (71-78%) [2,5], but it is less commonly associated with falling. Our case involves a patient with incidental traumatic spondylolisthesis of the axis due to a fall from a seated position. There are reports of incidental findings [6,7] but they still involve a motor vehicle accident or something equivalent. We will review the literature surrounding the fracture while emphasizing the importance of obtaining a thorough medical history in cases of in-home injuries to prevent serious complications.

## Case presentation

A 59-year-old male with no significant past medical history presented to the emergency department with complaints of intense, bilateral persistent neck pain due to a fall three days prior with a mild response to Motrin and steroids. Due to possible food poisoning, the patient became dehydrated from excessive diarrhea and vomiting. He admits passing out and waking up in his own vomit and hitting his head. The patient assumed that the neck pain arose from a sudden twisting of his neck and attributed the pain to that. The pain originated from the base of his skull, more left than right, extending to the scalp and the left shoulder. But after four days of intense pain limiting his ability to turn to rotate his neck and bend to tie his shoes, he came to the clinic to have his neck further evaluated.

His physical exam revealed guarded neck movement with limited range of motion, point tenderness at the occiput only, with normal skin contour. No neurological deficits or ecchymosis were noted, with intact carotid pulses bilaterally. All other findings were within normal limits.

**Figure 1:**
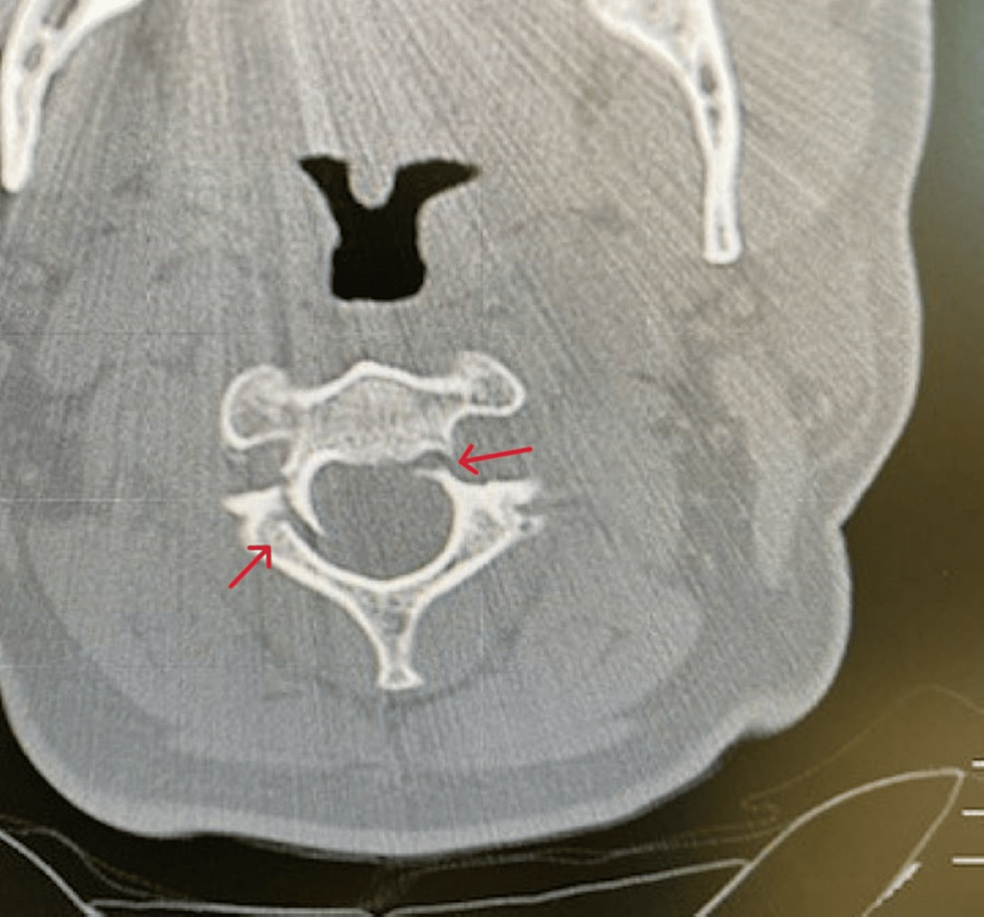
Non-contrast CT (transverse view) of the C2. Note the bilateral fracture separating the lamina and body.

**Figure 2:**
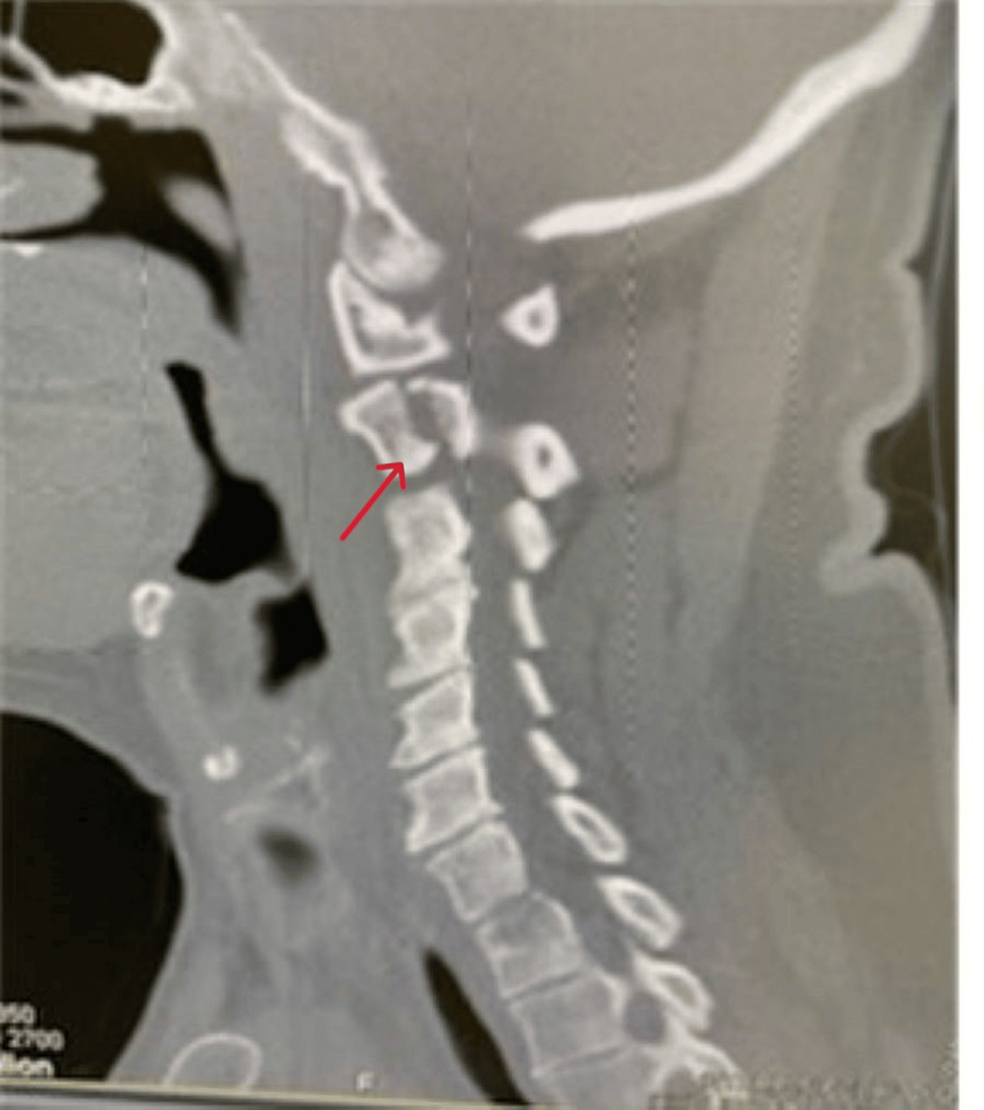
Non-contrast CT (sagittal view) of the cervical spine. Note the spondylolisthesis.

**Figure 3:**
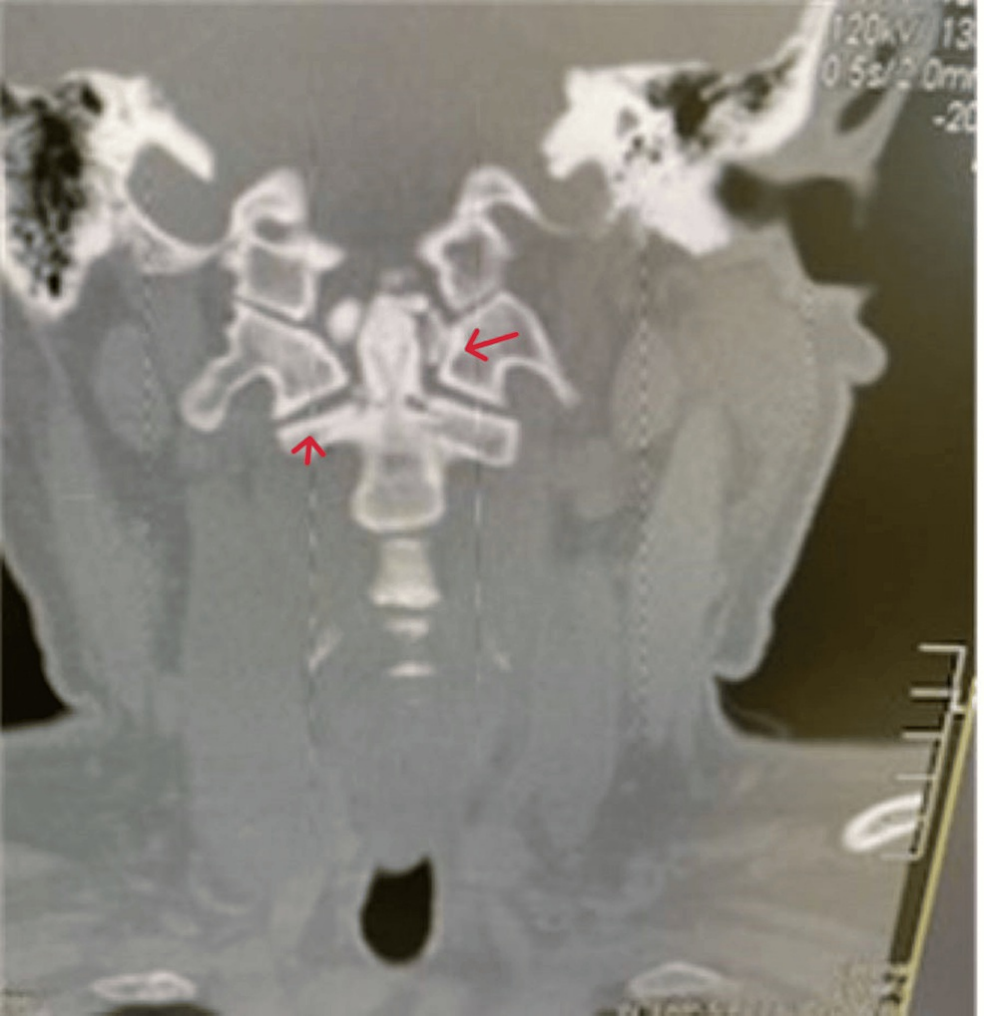
CT (coronal view) of the cervical spine showing spondylolisthesis.

**Figure 4:**
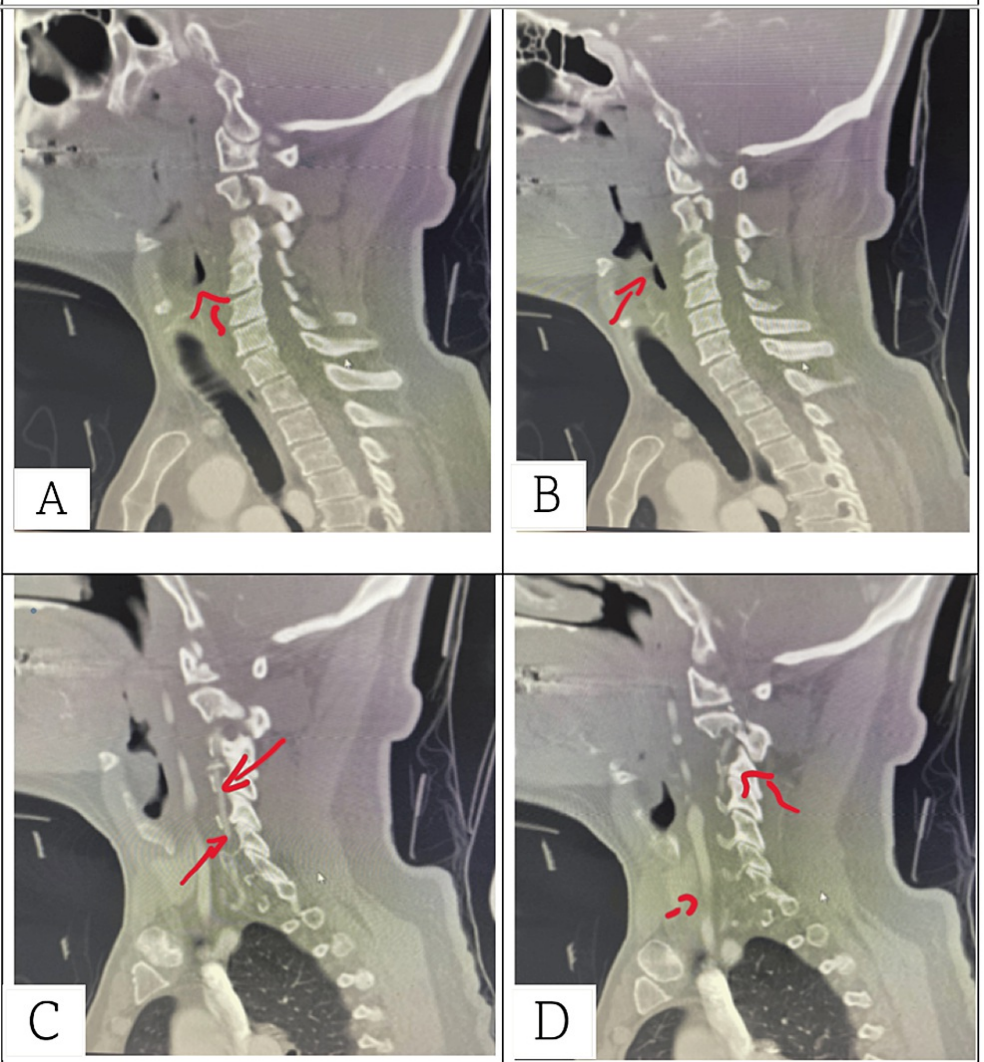
CT arteriogram (sagittal view) of the vertebral arteries with different sections labeled A, B, C, and D. The progression of the photos goes from internal to external. Image (A) shows the cervical spinal processes and the esophagus. Note the hypoechoic portion above the esophagus, indicating the left lateral side of the oropharynx. Image (B) shows slight external progression from the spinal cord where we see more of the oropharynx. This is to indicate the progression of CT going more lateral. Image (C) is more lateral, on the patient's right side. The hyperechoic portion, where we see the contrast, along the spinous processes is the vertebral artery. More anterior we see contrast uptake in the carotid artery. Note the lack of contrast outside of the spinal processes, indicating no perforation of the artery. Image (D) shows a more lateral view of the patient's neck. Note the contrast attenuation in the vertebral artery as well as no dispersion of the media.

Given the unresolved intensity of pain, a non-contrast CT of the cervical spine with coronal and sagittal views was obtained. CT (Figures 1-3) revealed a fracture of C2 with the pedicle on each side being separated by over 2 mm and mild right lateral displacement of about the same degree. Moderate intervertebral disc narrowing at C3-4, C4-5, C5-6, and C6-7 was shown. Articular sclerosis/hypertrophy was noted in each of those levels. The spinal canal was not compromised. Given that the patient had no symptomatology besides neck pain, he was given a hard cervical collar, a five-day course of steroids, and instructed to rest until he could see a neurosurgeon. A week later, the patient followed up with the neurosurgeon. The neurosurgeon identified the fracture as type I and decided to treat the patient conservatively with a hard collar for three months. Additionally, the neurosurgeon ordered a cervical CT angiogram (CTA) with IV contrast (Figure 4) with coronal and sagittal views to rule out arterial complications. CTA reported opacification of each vertebral artery with no evidence of dissection, leakage, or flow void. No evidence of vertebral artery abnormality post-C2 fracture was noted. A one-month follow-up CT scan was performed and showed similar results. The patient still has no neurological deficiency but does continue to have mild neck pain with movement. The patient continues wearing the brace. Four months after the incident, the patient still has residual pain but has fewer limitations to their neck, requiring less pain-relieving medications.

## Discussion

Traumatic spondylolisthesis of the axis describes a frequent area of breakdown in the pars interarticularis of the neural arch. This results in the detachment from the C2 vertebral body and the consequent forward slippage of the C2 over the C3 vertebral body. A hyperextension or hyperflexion force applied to the head can cause the upper cervical structures, including the odontoid peg and the body of the axis, to tilt backward or forward, respectively. Due to the facet joints being anchored by the body's weight, the articular processes are unable to follow this movement. This results in a bending moment being exerted on the vertebral body and pedicle of the axis, leading to failure at the weakest part of the bone, the interarticular portion of the neural arch. The injury mechanism can vary based on the additional force vector along the longitudinal axis. For instance, it can result in a hyperextension-distraction injury, which is common in "judicial hanging," or a hyperextension-compression injury, as seen in situations such as traffic accidents where passengers' foreheads strike the windshield. A distraction-type injury has the potential to completely disrupt all ligamentous structures, leading to severe instability and often fatal neurologic damage to the spinal cord at the C2 level [8].

However, later studies showed that this special fracture morphology accounts for ~10% of injuries observed in judicial hangings, yet the name persists [3]. Nearly 20% of acute cervical spine fractures involve the axis and the traumatic spondylolisthesis of the axis accounts for about 21% of all axis fractures. In actuality, "hangman's fractures" are the second most common type of axis fracture. The most common are odontoid fractures [5].

Suspicion for traumatic spondylolisthesis of the axis should be raised anytime there is a high-velocity hyperextension of the cervical spine, such as in cases of motor vehicle collisions or high-impact falls. A history of blunt trauma, osteoporosis, metastatic burden, or vitamin D deficiencies are considered risk factors [9]. According to one meta-analysis of retrospective studies [1], neurological impairment was only seen in 24% of all types of fractures. Neurological impairment scaled with the severity of the fracture. Physical exam findings include pain with palpation in the posterior portion of the neck, radiculopathy, myelopathy, or posterior fossa findings secondary to damage to the vertebral arteries such as difficulty speaking, swallowing, or maintaining their balance [10]. A strict neurologic exam is mandatory and should include cranial nerves, sensory and motor components, reflexes, and rectal tone [9].

The diagnosis of a hangman’s fracture can be made using X-rays, CT, or MRI to determine the severity of the fracture. There are several grading symptoms such as Effendi, Francis, and Levine and Edwards' system [11]. The most commonly used classification system is that of Levine and Edwards, which is a modified version of the original scheme described by Effendi. The version provided by Levine and Edwards focuses on the angulation measured between the inferior endplate of C2 and C3. This differs from the original classification scheme by Effendi where fractures were classified by visible fractures along with knowing the mechanism of injury. The new classification system may be easier with today's technology being able to measure with more accuracy. With Levine and Edward's classification, an anterior subluxation of C2 on C3 greater than 3 mm is a maker for C2 to C3 intervertebral disc disruption. While this newer classification may be more optimal for clinicians, it is important to note that this grading system does not apply to the pediatric population [12]. The classification system described above is presented in Table 1 [11].

**Table 1:** Levine and Edwards' classification system. Data obtained from [11].

Type	Description	Characteristics	Mechanism of injury	Associated injuries	Stability	Treatment
Type I	Minimally displaced fracture with ≤3 mm anteroposterior translation	≤3 mm anteroposterior translation; no angulation; C2/3 disc intact	Hyperextension and axial loading	Atlas fractures, specifically Jefferson, odontoid process, and posterior arch fractures	Stable	Collar immobilization
Type II	Displaced fracture with >3 mm anteroposterior translation	>3 mm anteroposterior translation; angulation ≤10°; vertical fracture line; C2/3 disc and posterior longitudinal ligament disrupted	Distractive flexion or compressive hyperextension	C3 anterosuperior compression fractures, C2 endplate avulsion fractures	Unstable	Halo immobilization or surgery
Type IIa	Type II fracture with minimal or no translation but marked angulation	Marked angulation >10°; horizontal/oblique fracture line; significant angular deviation without anterior translation; anterior longitudinal ligament intact	A similar mechanism to type II injuries	-	Unstable	Compression halo without traction (to avoid increasing fracture angulation)
Type III	Type II fracture with bilateral facet joint dislocation	Rare; distractive flexion	Surgery	-	-	Surgery

It's important to note that not all C2 hangman's fractures fit these classification systems. In a typical hangman's fracture, the separation of anterior and posterior elements of the C2 vertebra increases space for the spinal cord. Conversely, atypical hangman's fractures involve the posterior aspect of the C2 vertebral body, leading to a higher risk of spinal cord injury due to no increase in available space. This distinction between "typical" and "atypical" is outlined by Pinter et al. They describe typical fractures are those that have bilateral separation of the neural arch at C2 [13]. Atypical variants are, in short, those that do not follow that pattern. These variants include vertical fractures of the posterior aspect of the C2 body [13], isolated C2-C3 facet dislocation [14], injury to the capsular ligament of the facet joint and posterior spinous ligamentous complex [15], and pedicle fractures [16].

Vascular imaging should also be obtained as the main artery of concern is the vertebral artery. The anatomy is well documented in an article by Satti et al. [17], describing the vertebral artery dividing into four different segments. The vertebral artery originates from the subclavian artery. The first segment, marked as V1, is the extraosseous segment. This segment extends from the origin of the subclavian artery to the transverse foramen of the sixth cervical vertebra. This is the transition point where V1 becomes V2. The V2 segment traverses through the transverse foramen of C6 to C2. It is sheathed within the foramen as it continues to become the 3rd segment, V3, which runs extradurally. V3 exits the C2 foramen along a groove known as the sulcus arteriosus as it makes its way to the dura of the skull. Finally, the last transition point is here. The 4th segment, V4, originates from the dura at the lateral edge of the posterior atlanto-occipital membrane to their confluence on the medulla from the basilar artery [18].

In one case series, 15% of patients with C1 to C2 fractures had artery injuries, and type III fractures carry the highest risk of vascular compromise at 11% [17]. As with most injuries, understanding what the anatomy is will help guide clinical decisions. In addition to imaging, other labs and studies can be obtained as they pertain to the case. For example, a complete blood count that includes hemoglobin, hematocrit, and platelet counts along with coagulation studies such as prothrombin/partial prothrombin time and international normalized ratio (INR) will be utilized in trauma surgery. In minor cases, it may not be necessary to obtain labs, saving the patient from more medical fees. Management of traumatic spondylolisthesis of the axis is guided by a number of considerations. The first is the degree of stability. For assessing the angulation between C2 and C3, the end plate measurement method is more reliable than the posterior wall measurement method [19]. In terms of translation measurements, a plain X-ray offers a more dependable diagnosis than a CT scan [19].

Treatment options include nonoperative and operative procedures. The consensus on nonoperative treatment is the use of a semirigid immobilization using a cervical orthosis for six to 12 weeks for Effendi/Levine types I and II injuries [2]. Halo fixators are used when operative treatment is not possible or as a bridging measure until surgery is possible, although specifically for anterior surgical procedures. Halo traction is contraindicated in all hyperextension-traction injuries. For more severe cases of fractures, like type IIB and above, surgery is recommended. Internal fixation strategies should be considered in the following scenarios [9]: surgical fixation may be recommended under certain circumstances, including severe angulation of C2 on C3 (Francis II and IV, Levine II), disruption of the C2 to C3 disc space (Francis V, Levine II), anterior displacement of C2 greater than 50% on C3, failure to establish or maintain alignment with external immobilization, and nonunion following the use of external immobilization.

The expected prognosis for type 1 fractures with conservative management is 100% [11]. Complications include vertebral arteriovenous fistula, vertebral artery dissection, Brown-Sequard syndrome, and spinal cord injuries.

## Conclusions

In conclusion, this case report highlights the importance of a thorough medical history assessment in evaluating neck pain after falls. It presents a rare "hangman's fracture" from a minor fall, challenging the usual link with high-impact trauma. Detailed history-taking, including fall circumstances and pre-existing conditions, is crucial for accurate diagnosis and treatment. The patient’s successful management with a hard cervical collar reflects the appropriate approach for type I fractures.
